# Bone marrow stromal/stem cell-derived extracellular vesicles regulate osteoblast activity and differentiation *in vitro* and promote bone regeneration *in vivo*

**DOI:** 10.1038/srep21961

**Published:** 2016-02-25

**Authors:** Yunhao Qin, Lian Wang, Zhengliang Gao, Genyin Chen, Changqing Zhang

**Affiliations:** 1 Shanghai Jiaotong University Affiliated Sixth People’s Hospital, Shanghai, 200233, China; 2 Department of Anesthesiology, Affiliated Hospital of Guangdong Medical College, Yanjiang West Road, Guangzhou, 510120, China; 3 Shanghai Tongji University School of Medicine, Shanghai, 200029, China.

## Abstract

Emerging evidence suggests that extracellular vesicles (EVs) are secreted by diverse tissues and play important roles in cell-cell communication, organ interactions and tissue homeostasis. Studies have reported the use of EVs to stimulate tissue regeneration, such as hepatic cell regeneration, and to treat diseases, such as pulmonary hypertension. However, little is known about the osteogenic effect of EVs. In this study, we explore the role of bone marrow stromal cell-derived EVs in the regulation of osteoblast activity and bone regeneration. We isolated bone marrow stromal/stem cell (BMSC)-derived EVs through gradient ultracentrifugation and ultrafiltration, and tested the influence of the EVs on osteogenesis both *in vivo* and *in vitro*. The results indicated that EVs positively regulated osteogenic genes and osteoblastic differentiation but did not inhibit proliferation *in vitro*. Furthermore, we constructed an EVs delivery system to stimulate bone formation in Sprague Dawley (SD) rats with calvarial defects. We found that BMSC-derived EVs led to more bone formation in the critical-size calvarial bone defects. Moreover, we found that miR-196a plays an essential role in the regulation of osteoblastic differentiation and the expression of osteogenic genes. We anticipate that our assay using bone marrow stromal cell-derived EVs will become a valuable tool for promoting bone regeneration.

Extracellular vesicles (EVs) are a family of vesicles such as apoptotic bodies and exosomes^1^, which can be released by numerous types of cells such as dendritic cells^2^, reticulocytes^3^, tumor cells^4^, B cells^5^, T cells^6^, mast cells^7^ and epithelial cells^8^. Although their specific functions and mechanisms remain elusive, it is increasingly apparent that EVs play crucial roles in whole-body, cell-to-cell, and organ-to-organ communication. For example, EVs released by one cell at an organ site can spread and bind to other cells or to another cell type at another organ site through receptor–ligand interactions (e.g., antigen presentation)^6^. They can also travel through the body and attach and/or fuse with the target cell membrane to deliver surface proteins^9^ and cytoplasm to the recipient cells^10^. These vesicles can be internalized by the recipient cells by endocytosis^11^. In this way, the small vesicles can effectively communicate and deliver messages between cells and tissues, achieving systematic and integrative regulation of body homeostasis.

Under pathological conditions, EVs may be hijacked and cause pathogenesis, e.g., initiating tumor metastasis^12^. In contrast, EVs can also act to achieve orchestrated coordination at the system level by modulating tissue regeneration, repair and homeostasis in response to tissue erosion^13^. Evidence of this has been suggested by the observation that EVs of diverse origins contain numerous stemness-related bioactive molecules (RNA and proteins), and mounting evidence suggests that EVs are capable of transmitting stem cell phenotypes to recipient cells regulating injury response, stem cell maintenance, differentiation, self-renewal, and tissue regeneration^14^. For example, hepatic stellate cell-derived EVs inhibit hepatic fibrosis^15^ and mesenchymal stromal cell-derived EVs stimulate tissue resident stem cells, thus restoring the integrity and functionality of tissues^16^.

Large bone defects are a common, debilitating clinical condition^17^. Earlier studies based on animal models have suggested that local transplantation of BMSCs promote bone regeneration through unknown mechanisms^18^. BMSCs actively produce EVs, and BMSC-conditioned medium potently stimulates bone regeneration^19^. However, the potential role of BMSC-derived EVs in bone regeneration is unclear, and the possible underlying mechanisms have yet to be determined.

In the present study, we collected and isolated BMSCs from clinic. We examined the tentative role of EVs in bone regeneration and interrogated the underlying mechanisms. Our results show that human BMSC-derived EVs can enter the osteoblasts and deliver osteogenic miRNAs by endocytosis, thus modulating osteogenic gene expression and hence differentiation *in vitro*. Importantly, BMSC-derived EVs substantially promoted bone regeneration in SD rats with calvarial defects.

## Results

### BMSC-conditioned Medium Stimulates Osteoblastic Activity and Differentiation

**Figure 1:**
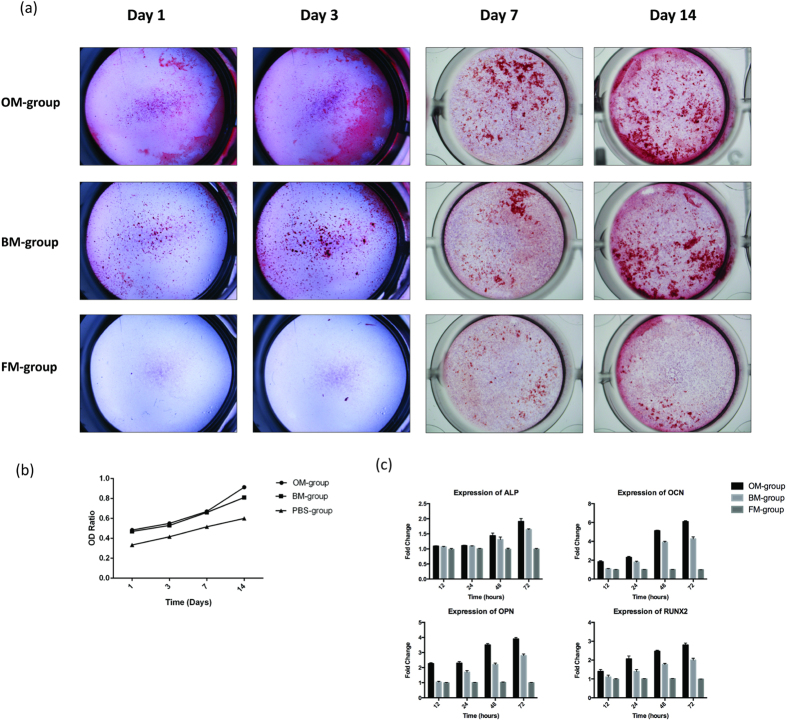
(**a**) The macroscopic Alizarin Red staining for the OM-group, the BM-group and the FM-group from Day 1 to Day 14 at each time point. (**b**) The OD ratio of Alizarin Red staining indicated that there was no statistically significant difference between the OM-group (0.84 ± 0.04) and the BM-group (0.82 ± 0.01) at day 14 (P = 0.97, P > 0.05), whereas there was a statistically significant differences between the BM-group and the FM-group (0.57 ± 0.03) (P = 0.001, P < 0.05). (**c**) The ALP expression increased by 2.2-fold and 1.6-fold in the OM-group and the BM-group compared to the FM-group, respectively. The OCN expression increased by 6.1-fold and 4.0-fold in the OM-group and the BM-group compared to the FM-group, respectively. The OPN expression increased by 4.1-fold and 2.9-fold in the OM-group and the BM-group compared to the FM-group, respectively. The RUNX2 expression increased by 2.8-fold and 2.1-fold in the OM-group and the BM-group compared to the FM-group, respectively.

To explore the tentative involvement of BMSCs in the recovery of bone defects *in vivo*, we first examined whether BMSC-conditioned medium (BM) could stimulate osteoblastic differentiation. As expected, the osteogenic medium (OM) induced robust differentiation of human osteoblasts. Similar results were observed for the BM group. In both groups, clear calcium deposits were observed by Alizarin Red staining on the first day and gradually increased as differentiation progressed through the 14-day procedure (Fig. 1a). The OD ratio of each group at the different time points also confirmed these results (Fig. 1b). Accordingly, the expression of osteogenic genes, including ALP, OCN, OPN and RUNX2, were highly induced in both the BM group and the OM positive control group, but not in the fresh osteoblast medium (FM-group) (Fig. 1c). Together, these results demonstrated that BMSCs conditioned medium is capable of stimulating osteoblastic differentiation, which confirms the findings of an earlier report^19^.

### Human BMSC-derived Extracellular Vesicles Support Osteoblastic Activity and Differentiation

**Figure 2:**
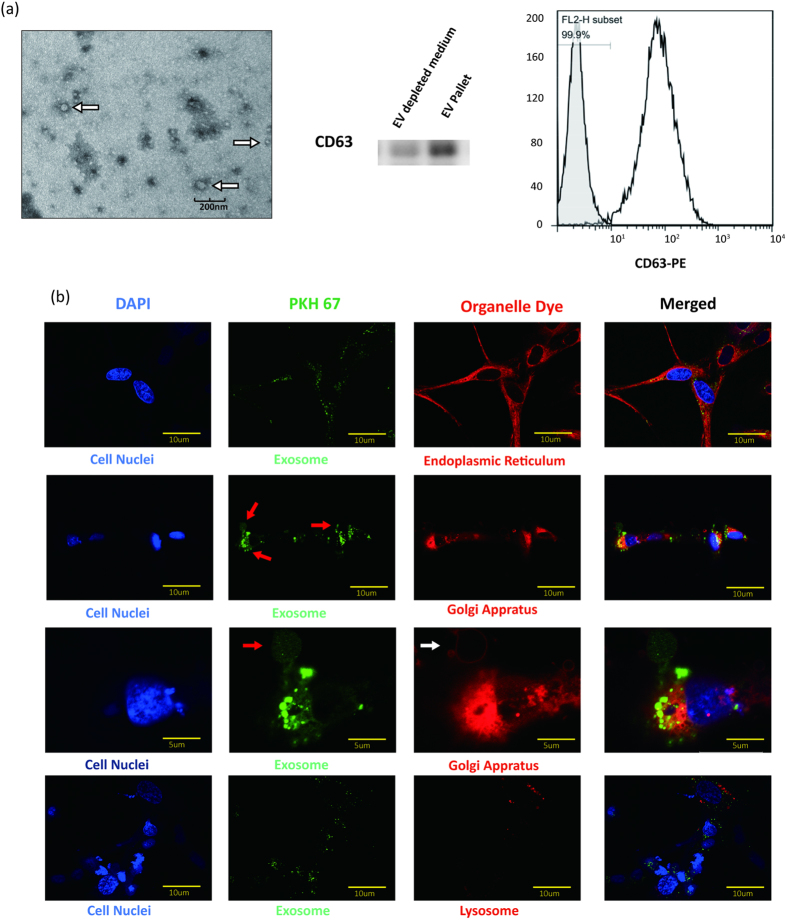
(**a**) A representative electron microscopic image of EVs derived from BMSCs (White arrow). Scale bar, 200 nm. The western blot of EV-depleted medium and EVs pallet also demonstrated that BMSC-derived EVs were isolated. For the FACS analysis, EVs (open trace) and negative control (filled trace) are shown. (**b**) Confocal fluorescence analysis was performed 4 h after incubation. The EVs were PKH67-labeled according to the manufacturer’s protocol (green fluorescence). The endoplasmic reticulum, Golgi apparatus and lysosomes were stained by ER-Tracker Red, Golgi-RFP and Lyso-Tracker Red. DAPI was used to stain the cell nuclei (blue fluorescence). Red arrows showed shallow green round-like staining indicating that the foreign EVs were degraded. The white arrow showed the round Golgi apparatus lumen.

Next, we examined whether EVs could stimulate osteoblastic differentiation and could potentially account for the pro-bone regeneration effect of BMSCs. The EVs according to a commonly used protocol^20^ (Fig. 2a, white arrows). The identity of the EVs was further confirmed by western blot analyses and flow cytometry (FACS) of CD63, a commonly used tentative EVs marker^14^,^21^. Then, we determined whether the BMSC-derived EVs were capable of fusing with and entering osteoblasts. Thus, we labeled the purified EVs with PKH67 (green), a stable green fluorescent cell membrane linker, and we labeled diverse cell organelles with organelle-specific dyes (red). We then added PKH67-stained EVs to the labelled osteoblasts. After a 4-hour incubation, the cells were imaged and analyzed by confocal microscopy. As shown in Fig. 2, the PKH67 linkers entered the cells and were present in the endoplasmic reticulum (ER-Tracker Red), the Golgi apparatus (Golgi-RFP) and the lysosomes (Lyso-Tracker Red) (Fig. 2b). These observations indicated that the EVs fused with the cells and were transferred to the Golgi apparatus, presumably by endocytosis, releasing their cargo along the way.

**Figure 3:**
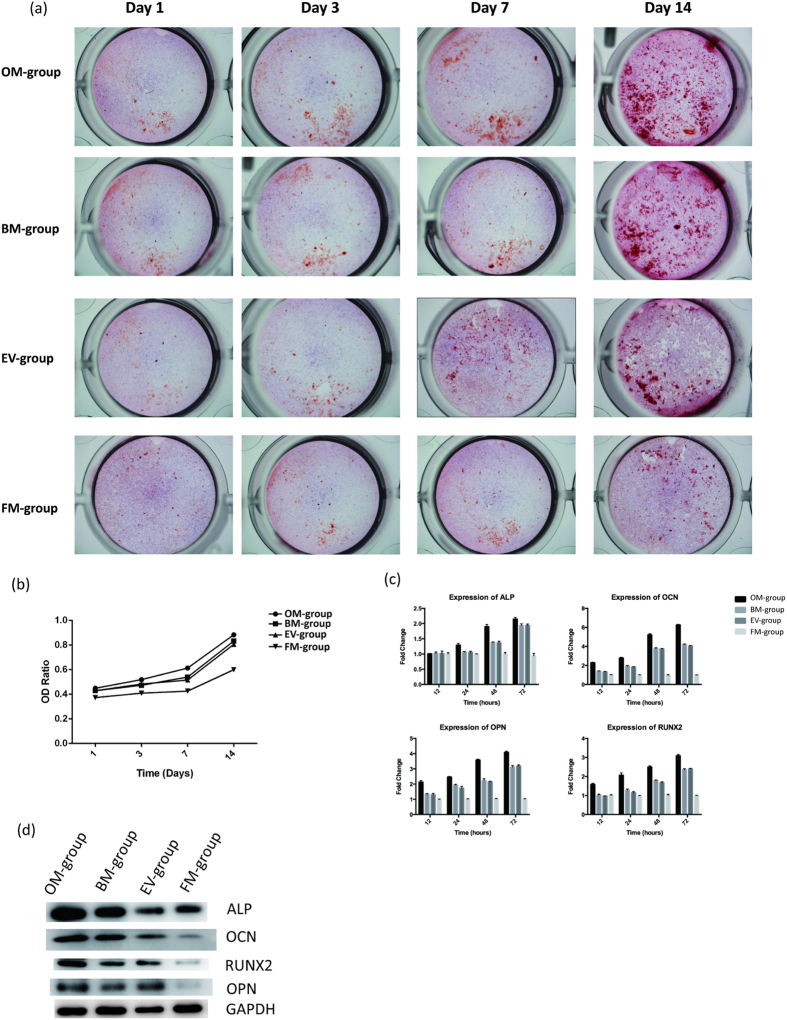
(**a**) The macroscopic Alizarin Red staining for the OM-group, the BM-group, the EV-group and the FM-group from Day 1 to Day 14 at each time point. (**b**) The OD ratio of Alizarin Red staining indicated that there was no statistically significant difference between the OM-group (0.83 ± 0.02) and the EV-group (0.78 ± 0.01) at day 14 (P = 0.09, P > 0.05), whereas there was a statistically significant difference between the EV-group and the FM-group (0.53 ± 0.03) (P = 0.001, P < 0.05). (**c**) The expression of ALP, OCN, OPN and RUNX2 increased by 1.9-, 3.8-, 3.2- and 2.4-fold, respectively, in the EV-group compared to the FM-group. (**d**) The western blot of ALP, OCN, OPN, and RUNX2 at day 14.

We then performed functional testing of the purified EVs by osteoblastic differentiation assays. As determined by Alizarin Red staining, there were substantial increases in calcium deposits in the EVs treatment group (EV-group), and the increases were comparable to those observed in the FM-group on days 1, 3, 7 and 14 (Fig. 3a), indicating the osteogenic potential of BMSC-derived EVs. Accordingly, the expression of osteoblastic markers and osteogenic genes, including ALP, OCN, OPN and RUNX2, were highly induced in the EV-group and were comparable to the OM-group. The OD ratio of each group at the different time points confirmed the results (Fig. 3b). Compared to the FM-group, the expression of ALP, OCN, OPN and RUNX2 increased by 1.9-, 3.8-, 3.2- and 2.4-fold, respectively, in the EV-group (Fig. 3c). Western blot analyses (Fig. 3d) further confirmed that ALP, OCN, OPN and RUNX2 were up-regulated in the EV-group, comparable to the FM group. Together, these results suggested that extracellular vesicles stimulated osteoblastic differentiation presumably by modulating the expression of osteogenic genes.

### Extracellular Vesicles have a Marginal Effect on Osteoblast Proliferation

**Figure 4:**
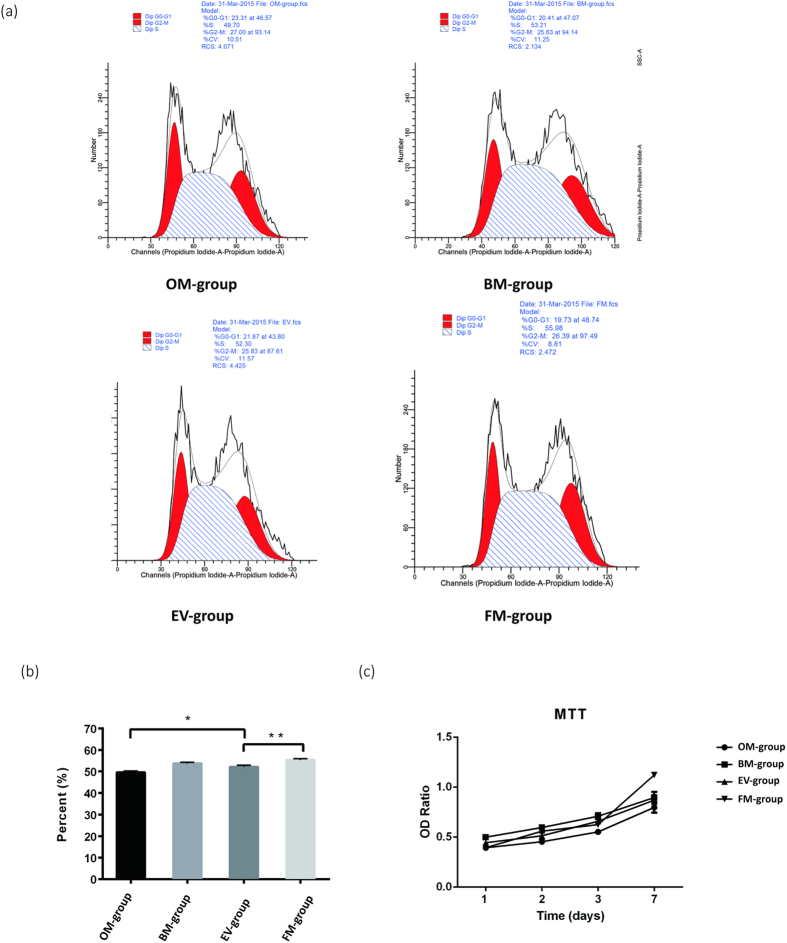
(**a,b**) FACS cell cycle analysis indicated that there was a statistically significant difference between the OM-group (S phase: 49.53 ± 0.68%) and the FM-group (S phase: 55.40 ± 0.51%), P = 0.001, P < 0.05. The EV-group (S phase: 52.16% ± 0.71%) showed statistical significance when compared to the FM-group (P = 0.001, P < 0.05). There was a statistically significant difference between the EV-group and the OM-group, P = 0.04, P < 0.05. There was no statistically significant difference between the EV-group and the BM-group (S phase: 53.76 ± 0.50%), P = 0.073, P > 0.05), *P < 0.05, **P < 0.001. (**c**) The OD ratio of the FM-group is 1.12 ± 0.02, the BM-group (0.89 ± 0.05) and the EV-group (0.87 ± 0.04) were higher than that of the OM-group (0.80 ± 0.05) but lower than that of the FM-group. There was a statistically significant difference between the FM-group and the EV-group (P = 0.028, P < 0.05). There was a statistically significant difference between the OM-group and the FM-group (P = 0.025, P < 0.05). There was no significant difference between the EV-group and the OM group (P = 0.121, P > 0.05). There was no significance difference between the EV-group and the BM-group (P = 1.000, P > 0.05).

Having established a role for EVs in osteoblast differentiation, we wondered whether they might also play a role in osteoblast proliferation. Thus, we performed cell cycle analysis by FACS (Fig. 4a,b) and cell proliferation analysis by MTT assay (Fig. 4c). For both the FACSs and the MTT assays, statistically significant differences were observed between the EV-group and the FM-group as well as between the EV-group and OM-group. However, the differences between the groups were rather small, suggesting that EVs may have only a marginal effect on osteoblast proliferation. Alternatively, this marginal inhibition of osteoblast proliferation could be secondary, perhaps due to EVs-mediated enhancement of osteoblast differentiation.

### Extracellular vesicles Regulate Osteoblastic Differentiation and Gene Expression through miR-196a

**Figure 5:**
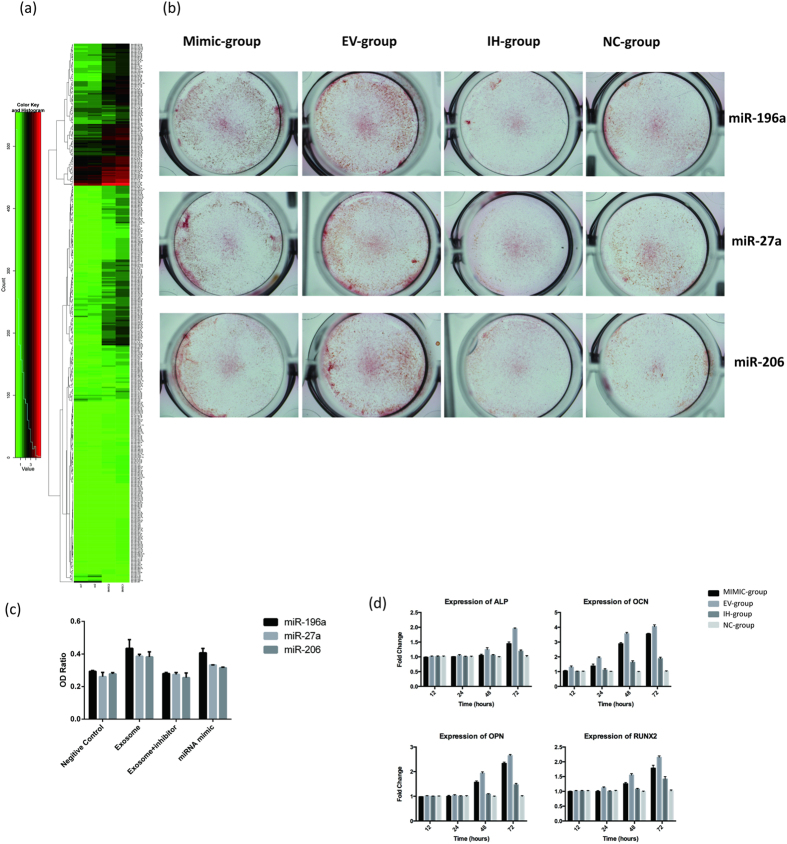
(**a**) The RNA sequencing of BMSC and EVs indicated that miR-196a, miR27a and miR-206 were highly enriched in EVs. (**b**) Alizarin Red staining of osteoblasts treated with miR-196a, miR-27a and miR-206 at 3 days. (**c**) The OD ratio of Alizarin Red staining indicated a statistically significant difference compare miR-196a Mimic-group (0.42 ± 0.01) to the miR-27a Mimic-group (0.36 ± 0.01, P = 0.001, P < 0.05), or to the miR-206 Mimic group (0.35 ± 0.01, P = 0.001, P < 0.05). (**d**) The expression of ALP increased 1.4-fold in the Mimic-group, 1.9-fold in the EV-group, and 1.2-fold in the IH-group compared to the NC-group. The expression of OCN increased 3.3-fold in the Mimic-group, 4.0-fold in the EV-group, and 2.0-fold in the IH-group compared to the NC-group. The expression of OPN increased 2.4-fold in the Mimic-group, 2.6-fold in the EV-group, and 1.3-fold in the IH-group compared to the NC-group. The expression of RUNX2 increased 1.7-fold in the Mimic-group, 2.2-fold in the EV-group, and 1.4-fold in the IH-group compared to the NC-group.

EVs transfer bioactive molecules including microRNAs to recipient cells regulating tissue repair and regeneration^14^. Thus, we extracted small RNAs and subjected them to miRNA/small RNA-Sequencing (Fig. 5a).

Among the most highly enriched miRNAs in the EVs were miR-196a, miR-27a and miR-206, three miRNAs critical for osteogenesis. Upon functional testing, all three miRNA mimics exhibited osteogenic effects as determined by Alizarin Red staining of calcium deposits, albeit to different extents, with miR-196a exhibiting the highest potency (Fig. 5b,c). Treatment with miRNA-specific inhibitors largely abolished e EVs mediated osteoblastic differentiation. At the molecular level, transfection of miR-196a induced the expression of ALP, OCN, OPN and Runx2 (Fig. 5d). As expected, co-treatment with a miR-196a inhibitor effectively attenuated the effects of the EVs and grossly reduced the expression of osteogenic genes, strongly suggesting that miR-196a partially mediates the EVs dependent osteogenic effects. Nonetheless, the expression levels of the osteogenic genes in the inhibitor and EVs co-treatment group were still higher than that in the non-treatment group, suggesting that there are other factors such as miR-27a and 206a in the EVs regulating these processes.

### Extracellular vesicles Stimulate Bone Regeneration *in vivo*

**Figure 6:**
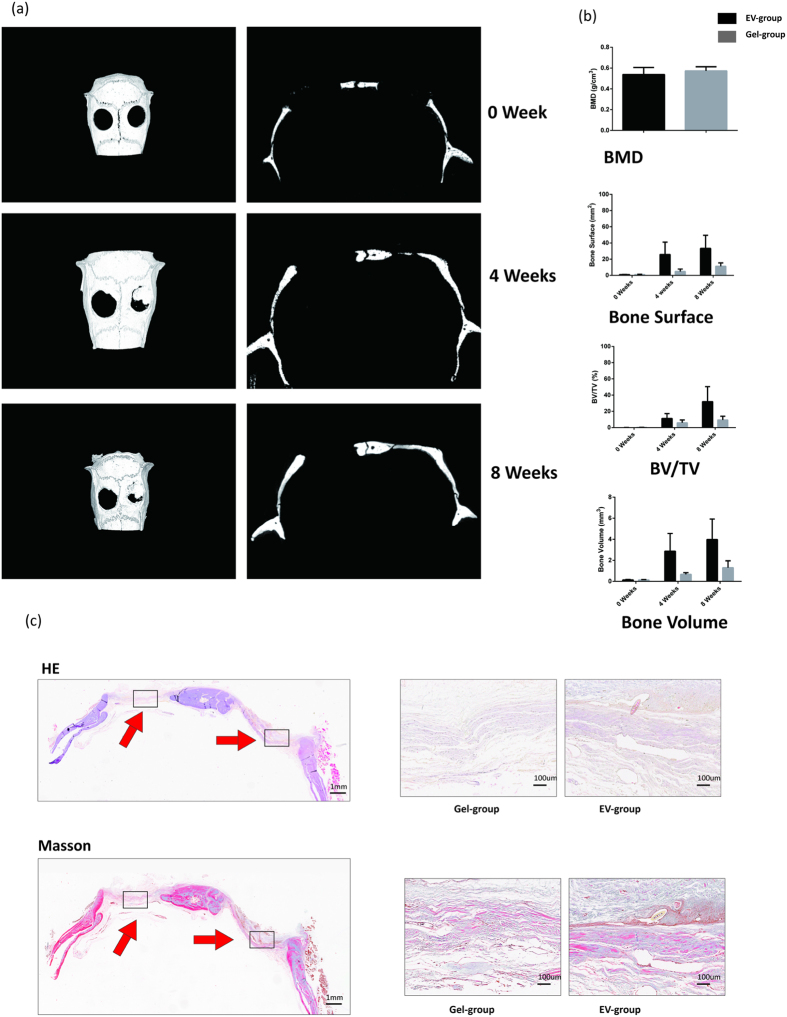
(**a**) The EV delivery system improved bone repair. Micro-CT of live mice from 0 to 8 weeks after surgery. The EV-group is the right defect and the Gel-group is the left defect (**b**) Bone surface (8 weeks, EV-group 32.8 ± 16.9 mm^2^, Gel-group 11.3 ± 4.0 mm^2^, P = 0.041, P < 0.05, n = 6). Bone volume (8 weeks, EV-group 4.0 ± 1.9 mm^3^, Gel-group, 1.3 ± 0.7 mm^3^, P = 0.042, P < 0.05, n = 6). Bone Volume/Tissue Volume (8 weeks, EV-group 32 ± 17.4%, Gel-group 9 ± 4.4%, P = 0.043, P < 0.05). BMD (8 weeks, EV-group 0.53 ± 0.06 g/cm^3^, Gel-group 0.49 ± 0.19 g/cm^3^, P = 0.627, P > 0.05). (**c**) HE and Masson staining revealed that the EV-groups were characterized by complete repair and the Gel-groups were characterized by moderate repair.

Because BMSC-derived EVs exhibited a strong osteogenic potential *in vitro*, we explored the EVs’ clinical relevance by performing *in vivo* functional tests. To that end, we generated a pair of 5-mm critical-sized calvarial bone defects in SD rats (n = 6). In the left holes, hydrogel without EVs was applied to the Gel-group and, in the right holes, hydrogel + EVs was applied to the EV-group. From 0 to 8 weeks post-operation, micro-CT was used to track the newly formed bone within the defects. As shown in Fig. 6, whereas the gel alone resulted in only minor bone regeneration, human patient BMSC-derived EVs containing gel accelerated the bone regeneration and showed clear enhancement of the repair as determined by a semi-quantitative analysis (Fig. 6b). At the end of the 8-week period, we sacrificed the animals and performed histological examinations. The amount and area density of neo-formed bones were both significantly increased in the EV-group compared to the Gel-group. Consistently with these results, HE and Masson staining supported the finding that EVs promote bone regeneration *in vivo* (Fig. 6c).

## Discussion

MSCs are known to stimulate tissue regeneration; however, the mechanism by which this effect occurs has not yet been elucidated. Among numerous attempts to identify the pro-regenerative mechanism, EVs released from MSCs have drawn much attention recently. Studies have shown that MSC-derived EVs are able to improve recovery in animal models of experimentally induced acute renal injury^22^ and myocardial ischemia/reperfusion injury^23^. In addition, Osugi *el al*.^19^ have found that MSC-conditioned medium greatly promoted bone regeneration in animal models. However, the exact mechanism is not yet known. Therefore, we explored the role of BMSC-derived EVs in bone both *in vivo* and *in vitro.*

According to our confocal observation, the shallow green round-like staining (red arrow) indicated the foreign EVs were degraded, and perfectly fitted to the round Golgi apparatus lumen (white arrow) (Fig. 2c). Therefore, these results indicated that exogenous EVs successfully entered osteoblasts and were degraded by the Golgi apparatus. We provided some evidence that the Golgi apparatus play important role in the degradation process of the BMSCs derived EVs. However, we still know little about the exact mechanism and location of EVs degradation. Claudia Campanella *et al.*^24^ demonstrated that heat shock protein 60 (Hsp60) located in the tumor cell membrane and the Golgi apparatus plays an important role in trafficking exogenous EVs into the Golgi apparatus. On the other hand, there are also other studies suggesting that EVs are degraded by lysosomes. Lydia Alvarez-Erviti *et al.*^25^ have found that the release of alpha-synuclein from EVs dramatically increases in the presence of lysosomal dysfunction in SH-SY5Y cells, indicating an important role for the lysosome in the process of EV degradation. The controversial observations may depend on whether EVs are endogenous or exogenous.

BMSC-derived EVs release their cargo and stimulate osteogenic gene expression, osteoblast differentiation *in vitro* and bone regeneration *in vivo. In vitro* experiments indicate that RUNX-2, ALP, OCN and OPN are highly up-regulated in cells treated with EVs. However, the proliferation of osteoblasts is not dramatically inhibited by the EVs.

Therefore, we explored the possible mechanisms by which EVs might promote bone regeneration. EVs are recognized as important mediators of intercellular communication, especially in the immune system. It has been proposed that EVs can act as a delivery system for the transfer of genetic information (mRNA and microRNAs) or can shuttle proteins to recipient cells^26^. Valadi *et al.*^27^ have found that miRNAs are highly enriched in EVs. Chen L *et al.*^15^ have demonstrated that miR-214 is delivered by EVs in hepatic fibrosis. Alvarez-Erviti *et al.*^28^ have delivered siRNA to the brain and successfully relieved Alzheimer disease through exosomes, a subtype of EVs. Therefore, we hypothesize that RNAs play a key role in the regenerative effect of EVs. We compared the miRNAs in BMSCs and EVs by RNA sequencing. The results showed that three osteogenic-related miRNAs, miR-196a, miR-27a and miR-206, were highly enriched in BMSC-derived EVs. Alizarin Red staining and qRT-PCR indicated that osteoblasts treated with miR-196a exhibited the best osteogenic activity.

Thus, we decided to explore whether BMSC-derived EVs may promote bone regeneration *in vivo*. Li *et al.*^29^ have loaded hydrogels with miRNAs and achieved bone regeneration in SD rats with calvarial bone defects. Therefore, we designed a system composed of a biodegradable hydrogel and EVs. The system was implanted into SD rats with a pair of 5-mm calvarial defects. The follow-up micro-CT and histological examination demonstrated that the EVs significantly improved bone regeneration, which resulted in the repair of the defect. In contrast, there was only partial bone repair in the control groups during the same time period.

In addition, EVs refer to an array of vesicles such as apoptotic bodies, microvesicles, ectosomes, exosomse and shedding vesicles. Therefore, it is important to establish specific and reliable approaches to precisely discern and identify subtypes of vesicles. Wu, *et al.*^21^ distinguished exosomes from EVs through electron microgsocpy size analysis and developing novel FACS approach to anaylize the protein on exosomes. These new approaches will help to indentify each subtypes of EVs and explore their specific roles in cell to cell communication.

## Conclusion

BMSC-derived EVs promote osteogenic function through the regulation of osteoblast differentiation and expression of osteogenic genes *in vitro*. The hydrogel + EV delivery system significantly enhances bone formation *in vivo*. Furthermore, the small vesicles may regulate bone regeneration through miRNA-196a. In our studies, we also found that exogenous EVs directly enter the Golgi apparatus rather than the lysosomes. The BMSC-derived EV-based strategy used in this study may be a valuable tool for improving tissue regeneration by simultaneously regulating multiple signaling pathways.

## Method

### Cell Culture

Human BMSCs were isolated according to current protocols. The use of all human samples was approved by and carried out in accordance to the ethical committee of the Shanghai No. 6 Hospital, Shanghai, China. Briefly, BMSCs were obtained from the bone marrow of 2 donors (aged 24 and 30. The donors are fully informed and signed informed consents) Cells from passages 3 to 5 were used in the experiments. BMSCs were cultured with α-MEM (Catalog: 12571071, Gibco, USA) and 10% fetal bovine serum (Catalog: 10099141, Gibco, USA).

Human osteoblasts (hFOB 1.19) were obtained from the Chinese Academy of Science and were cultured under standard cell culture conditions (a 37 °C, humidified, 5% CO_2_ environment) in Dulbecco’s-modified Eagle’s medium: nutrient mixture F-12 (DMEM/F-12 medium, Catalog: 12400024, Gibco, USA) supplemented with 10% fetal bovine serum.

The osteoblasts were continuously treated with EV (5 μg/ml in all *in vitro* experiments, EV-group), BMSC conditioned medium (BM-group), osteogenic medium (OM-group, Osteolife Compelet Osteogenesis Medium, Catalog: LM-0023, Lifeline, USA) and fresh osteoblast culture medium (FM-group) to explore the influence of EVs on osteoblasts.

### Extracellular vesicle Isolation

EVs were prepared from the supernatant of BMSCs by differential centrifugation according to current protocols^28^. Briefly, BMSC-conditioned medium cultured for 24 hours was harvested, centrifuged at 500 g for 30 min to eliminate cells and at 16 500 g for 20 min, followed by filtration through a 0.22 μm filter to remove cell debris. EVs were pelleted by ultracentrifugation (Beckman Ti70 rotor, USA) at 120 000 g for 120 min. EVs were measured for their protein content using a BCA Protein Assay Kit (Pierce, USA).

### Electron Microscopy

Isolated EVs were fixed in 2% PFA in 200 mM phosphate buffer (pH 7.4). Fixed EVs were dropped onto a formvar-carbon–coated grid and allowed to dry at room temperature for 20 min. After washing in PBS, the EVs were fixed in 1% glutaraldehyde for 5 min, washed in water and stained with saturated aqueous uranyl oxalate for 5 min. Samples were then embedded in 0.4% uranyl acetate and 1.8% methylcellulose and incubated on ice for 10 min. The excess liquid was then removed. The grid was dried at room temperature for 10 min and viewed at 20,000× magnification using an electron microscope (Philips CM 120, Netherlands).

### Flow cytometry

30 μg EVs were incubated with CD63 bead (Catalog: 10606D, ThermoFiher Scientific, USA) and IgG bead in negative control (Catalog: 130047501, Miltenyl Biotec, USA) according to the manufacture’s introduction. Then, EV-coated beads were washed twice, incubated in 1% human serum at 4 °C for 15 min, washed twice and incubated with CD63-PE antibody (ab77227, abcam, USA), and washed and analysed.

### Confocal Microscopy

Purified EVs were labelled by PKH67 green fluorescent cell linker (Catalog: PKH67GL-1KT, SigmaAldrich, USA) according to the manufacturer’s instructions and were then added to hFOB1.19 cells in culture medium. A total of 2.5 × 10^5^ osteoblasts were seeded in each well of a 24-well plate. EVs were introduced when cells reached 70–80% cell confluence. After 4 hours of incubation, the cells were washed three times in PBS and then fixed with 4% PFA. The endoplasmic reticulum (ER-Tracker Red, Catalog: E34250, Life Technologies, USA), the Golgi apparatus (Golgi-RFP, Catalog: C10593. Life Technologies, USA) and the lysosomes (Lyso-Tracker Red, Catalog: L12492, Life Technologies, USA) were stained according to the manufacturer’s instructions. The cell nuclei were stained by DAPI as previously described.

### Staining of Alizarin Red

Cells were fixed in 70% ice-cold ethanol for 30 min and rinsed with double-distilled H_2_O and then stained with 40 mM Alizarin Red S (Catalog: 130223, Sigma Aldrich, USA), pH 4.0, for 15 min with gentle agitation. Cells were rinsed five times with double-distilled H_2_O and then rinsed for 15 min with 1 × PBS while gently agitating. Images were captured at each time point (1, 3, 7, and 14 days) with a light microscope.

### Quantitative RT-PCR (qRT-PCR)

The expression levels of osteogenic genes were evaluated by qRT-PCR for marker genes including runt-related transcript factor-2 (RUNX2), alkaline phosphatase (ALP), osteopontin (OPN) and osteocalcin (OCN) in hFOB1.19 cells. OCN forward: GCCCTCACACTCCTCGCCCTATT, OCN reverse: GGGTCTCTTCACTACCTCGCTGCC. OPN forward: ACAGCATCGTCGGGACCAGACTCGT, OPN reverse: GGTAGTGAGTTTTCCTTGGTCGGCG. ALP forward: GGCAGCTTGACCTCCTCGGAAGACA, ALP reverse: AGCATGGGGGCCAGACCAAAGATAG. Runx2 forward: CCCCTCCTACCTGAGCCAGATGACG, Runx2 reverse: AAGGGCCCAGTTCTGAAGCACCTGA. Beta-actin forward: CGGGAAATCGTGCGTGACAT, Beta-actin reverse: GGACTCGTCATACTCCTGCTTGC.

### Cell Proliferation Assay

hFOB1.19 cells were harvested, centrifuged and washed 3 times with PBS. Then, the cells were suspended and fixed in 70% ethanol at 4 °C overnight. Fixed cells were washed twice with PBS and then suspended in 500 μl PBS containing 50 μg/ml PI, 100 μg/ml RNase A, 0.2% Triton X-100 and incubated for 30 min. Then, the cells were loaded on FACS. The MTT (Molecular Probes) assay was performed according to the manufacturer’s instructions, and the absorbance was measured at 450 nm with a microplate reader (MK3, Thermo Scientific, USA).

### Western Blot

Cells were harvested, sectioned and lysed in 10 mM Tris buffer, pH 7.4, containing 0.1% SDS, a protease inhibitor cocktail and DNase. Primary antibodies used for western blot analysis were rabbit anti-human ALP antibody (Catalog: ab95462, abcam, USA), rabbit anti-human OCN antibody (ab133612, abcam, USA mouse anti-human OPN (Catalog: ab69498, abcam, USA) and mouse anti-human RUNX-2 antibody (Catalog: ab23980, abcam, USA).

### RNA Sequencing

For analyzing the miRNA expression between EVs and BMSCs, total RNA of the samples was extracted using the RNeasy Mini Kit (Catalog: 74034, QIAGEN, USA). RNAs ranging from 18–30 nt were isolated by electrophoresis. Isolated RNAs underwent terminal repair by addition of a 5′-adapter and a 3′-adapter. Then, the RNAs were amplified and cDNAs were prepared. Raw small RNA sequence data were obtained by using Illumina HiSeq^TM^ 2500. Low quality data were filtered and adapters were removed to generate clean data. The clean data were prepared for genome mapping, exon annotation, ncRNA annotation and novel miRNA prediction. Here, the ncRNAs were further used for miRNA profiling to compare the different expression levels in BMSCs and EVs. miRNA networks were predicted by using Gene Ontology, KEGG, Pfam, and Interpro.

### microRNA (miR) Intervention

All the inhibitors, mimics and the negative controls for miR-196a, miR-27a and miR-206 were purchased from Ruibo (Guangdong, China). The miRNAs were prepared according to the manufacturer’s instructions. Cells were treated with negative control (NC group), EV (EV group), miRNA mimic (Mimic group) or EV + inhibitor (IH group).

### Animal Models

SD rats (n = 6, age = 8 weeks) were obtained from the Shanghai No. 6 Hospital affiliated with Shanghai Jiaotong University. All animal experiments were approved by and conducted in accordance with the committee guidelines of the Shanghai Jiaotong University School of Medicine, Shanghai, China and met the NIH guidelines for the care and use of laboratory animals. To study the influence of the EV system on bone repair, a pair of 5-mm calvarial defects were generated in each SD rat. The delivery system was composed of HyStem-HP hydrogel (Catalog: GS315, Glycosan Biosystems, USA) and EVs. A total of 100 μg of EVs in 50 μl PBS were mixed in hydrogel according to the manufacturer’s instructions^29^. The left defects were treated with hydrogel for the Gel-group, and the right defects were treated with hydrogel + EV (100 μg/per mice) for the EV-group. At 8-weeks post-implantation, bone regeneration in the defects was evaluated and further analyzed by histological techniques as described below.

### microCT

To track new bone formation, calvarial bones of anesthetized rats were scanned and analyzed using microCT (Bruker micro-CT system, Germany) every 4 weeks post-surgery.

### HE and Masson

At 8-weeks after implantation, the implants were harvested. For histological analysis, the implanted samples were fixed in 10% neutral buffered formalin overnight and embedded into paraffin. Sections were deparaffinized and stained with hematoxylin and eosin (H&E) or Masson’s Trichrome (MT) following previously described procedures. The results were observed under a light microscope (Leica SCN 400).

### Statistical Analysis

Alizarin staining, western blot, qRT-PCR, FCS, MTT and miRNA intervention were repeated at least three times and data are presented as the mean ± SD. Differences among the results of *in vivo* and *in vitro* studies were assessed using the Bonferroni’s multiple comparison tests, and statistical significance was analyzed using SPSS 20.0 software (IBM). P < 0.05 was considered statistically significant.

## Additional Information

**How to cite this article**: Qin, Y. *et al.* Bone marrow stromal/stem cell-derived extracellular vesicles regulate osteoblast activity and differentiation *in vitro* and promote bone regeneration *in vivo. Sci. Rep.* **6**, 21961; doi: 10.1038/srep21961 (2016).
